# Hospital and Institutional News

**Published:** 1916-02-05

**Authors:** 


### HOSPITAL AND INSTITUTIONAL NEWS.

### WILL EVERY READER PLEASE HELP?

**In the interests of this country, the Empire, and our Allies the Government have intimated an immediate reduction in the importation of paper, paper pulp, and other materials for paper-making. This necessary action of the Government makes it imperative that not a single copy of "The Hospital" shall be wasted, to which end returns must be stopped. This can only be accomplished by readers of "The Hospital" assisting us to the utmost to help the Government and our Allies, by giving an order to their newsagent or bookseller to-day for their weekly copy of "The Hospital." May we rely upon them thus to join hands with the Editor in helping to shorten the war? Every reader who at once places an order with his newsagent will co-operate to the full in this matter, and will render the completest possible help to our country and our Allies.**

### A LOSS TO CHARING CROSS HOSPITAL.

By the death of Lieutenant-Colonel Stanley Boyd, R.A.M.C., T.F., Charing Cross Hospital, like the Westminster by the death of Colonel Stonham, loses its senior surgeon, and both of them were, curiously enough, University College Hospital men of much the same date. In other respects there was not much resemblance between them, except that both were eminent surgeons. Mr. Stanley Boyd, as he was best known to the London Hospital world, was a tower of strength in council; his urbanity and knowledge of the world were of the greatest value to his adopted school on many occasions: and his popularity with students and with his private patients was firmly established. Mr. Boyd was a prolific writer, and his polished style and breadth of view made him much sought after as a contributor to "systems" and encyclopædias of surgery. He had been connected with several other London hospitals besides Charing Cross, including the Brompton Hospital for Consumption, the Paddington Green Children's Hospital, the New Hospital for Women, and several cottage hospitals in the London area. His wife, the late Mrs. Florence Boyd, who died four or five years ago, was one of the best-known medical women of the day, and was for many years on the staff of the Royal Free Hospital.

### A SECRETARY'S SALARY REVISED.

It is pleasant to record the decision of the committee of management of the Royal Portsmouth Hospital to raise the salary of the secretary, Mr. Wagstaff, by two annual increments, from £350 to £450 a year. Mr. Wagstaff was lately a candidate for the post of secretary to the Norwich Hospital where the salary is £450. The finance committee at Portsmouth, however, regarded the prospect of losing his services as unfortunate, and after an interview Mr. Wagstaff withdrew his candidature. He has been at Portsmouth Royal Hospital for ten years. It is gratifying to record each instance of the growing recognition of the need for attracting the best men to these secretarial posts, and for completing the process of advancement which the increased responsibility of administrative work has made a distinct feature of the past few years.

### SOME ABSURDITIES OF IGNORANCE.

That the need for ensuring and maintaining the highest standard in administrative posts is still too much unrecognised can be seen from one of the speeches made at a recent meeting at Portsmouth which sanctioned the increase in the salary of Mr. Wagstaff, secretary of the Royal Portsmouth Hospital. The speaker, who uttered the only dissentient voice, professed to represent the Hospital Saturday Committee, and urged the extraordinary objection that, as the secretary's salary was a great deal more than that of the house surgeon, it ought not to be increased. The want of apprehension revealed by this remark is too apparent to require comment. When we add that this statement was followed by another to the effect that the gentleman who held the corresponding position at the asylum only received £200 a year, and therefore any higher salary elsewhere was not to be thought of, it is patent that Hospital Saturday Committees or their representatives have sometimes a good deal to learn. If the best men are to be attracted or retained, salaries worthy of their work must be given to them. Hospital work is too complex for the second best man at a third-rate salary. The committee of the Royal Portsmouth Hospital are to be congratulated on the judgment which they have shown.

### AN INSTITUTIONAL PERSONALITY.

By the death of Colonel C. Stonham, C.M.G., F.R.C.S., the Westminster Hospital loses its senior surgeon and the London institutional and surgical worlds a striking personality. Educated at University College Hospital, Mr. Stonham, as he was in civil life, held several appointments at that hospital before his election as assistant surgeon at Westminster. A series of sudden and early deaths among the surgical staff resulted in his advancement to senior surgeon at a very unusually early age, some fifteen or sixteen years ago. To great height and to an extraordinarily slender frame he superadded a long lean face and a long narrow head, which gave him an appearance of ferocity; and a brusque manner confirmed this impression in many people's minds. As a matter of fact, no one could be more considerate of a suffering patient in hospital than Mr. Stonham, though it was not everyone who had discernment enough to appreciate this fact. To colleagues, juniors, and nurses he was on occasions not very conciliatory, and scores of stories are told at the Westminster of his sayings and doings. He examined for many years at the Apothecaries' Hall, where he had a (quite undeserved) reputation among the candidates for severity. Besides his marked ability as an operator, Mr. Stonham was an ornithologist of high repute.

Not many years ago he published a monograph on British Birds in twenty parts, which was a monument of patient observation and encyclopædic knowledge of the subject. He was decorated for his services in the South African War as major commanding the Yeomanry Field Hospital, and was afterwards for several years in command of the London Mounted Brigade Field Ambulance of the Territorial Force.

### MISS EVA LUCKES.

Our readers will share our satisfaction at being able to report a continuance in the progress of Miss Lückes towards recovery.

### ANTI-ENTERIC INOCULATION.

Until a very recent date the vaccine used in inoculating officers and men of the British Army against enteric fever has contained typhoid bacilli alone, and therefore, as explained in our article last week (p. 385), has protected the soldiers who submit to inoculation against typhoid fever only, not against the two paratyphoid fevers. This state of affairs is now altered, for it is stated that from the New Year all stocks of the old vaccine are to be made obsolete, and a new vaccine, made from the paratyphoid bacilli as well as from the typhoid bacillus, is to be used in future. This change of policy is somewhat significant, inasmuch as in November last Sir William Leishman, at the Royal Society of Medicine, expressed himself as strongly opposed to the use of mixed vaccines. He also declared that paratyphoid fever was then practically extinct, a view with which other speakers did not concur. The new policy of the Army Medical Service is a victory for Professor Castellani and other vaccine experts, and is evidence that the views on paratyphoid of the civil medical officers have at last prevailed over the opposition of Sir W. Leishman. It may be hoped that this year the incidence of paratyphoid may be as low as that of typhoid has hitherto been.

### A MEDICAL INSPECTORS' SALARY.

At a recent meeting of the Walsall Town Council a recommendation of the General Purposes Committee that the salary of the school doctor should be increased from £350 to £400 was defeated by a narrow majority. From the report of the discussion one gathers that this official is under thirty, and that some of the Council were influenced by the feeling that the Royal Army Medical Corps should have the first claims on his services, and that the children could be sufficiently looked after by the M.O.H. and those trained nurses who are available. Notwithstanding the importance of school medical inspection, we cannot help agreeing with this view. All young doctors who are physically fit should be in the R.A.M.C.; and if no women doctors or men of over military age are available for posts such as that at Walsall, the Medical Officers of Health must do as best they can with the help of trained nurses. It would be intolerable if junior medical officers, institutional or municipal, were to regard the pay of temporary lieutenants in the R.A.M.C. (which is nearly £500 a year) as setting a standard by which their own emoluments ought to be adjusted.

### HACKNEY RED CROSS HOSPITAL.

The Lord Mayor and the Lady Mayoress visited Clapton on January 29 for the purpose of opening the new Hackney and Stoke Newington Red Cross Hospital. This auxiliary hospital has been established in Stormont House, Downs Park Road, Clapton, and, with the exception of the usual War Office grant, will be conducted mainly by voluntary work and subscriptions. The building has been granted for the purposes of a hospital by the London County Council on a purely nominal rental. There will be accommodation in the hospital for from thirty to forty beds, and the War Office has been notified that the first thirty beds are already available for the reception of patients.

### A RECORD DISTRIBUTION.

In more ways than one the special meeting of the board of delegates of the Hospital Saturday Fund, held last week, constituted a record. The business was transacted in the record time of thirty minutes, and the amount distributed was a record also, being £7,000 more than that of last year, and £1,000 more than the sum granted in 1911. The report of the Distribution Committee, which was submitted by Mr. Dilley and unanimously adopted, stated that the total receipts, General Fund, amounted to £36,796. A summary of the awards shows that these reached a total of £35,040, and that 223 institutions received grants. These facts are very encouraging, and speak as well for the administration of the Fund as they do for the generosity of its constituent members.

### THE CENTRAL MIDWIVES BOARD'S ARBITRARINESS.

In The Hospital for March 6, 1915, we drew attention to the sudden and arbitrary decision of the Central Midwives Board to refuse their recognition to lectures in the London district not connected with institutions. We pointed out at the time the gross unfairness of, and the inconvenience likely to arise from, this decision. Another instance has now occurred of a sweeping change in the Board's regulations made without any consultation of those engaged in the training of midwifery pupils. In the early part of last year the Board passed a resolution extending the minimum period of training for pupil midwives from three to six months. This resolution was embodied in regulations which also increased the minimum number of lectures to be attended from fifteen to twenty, and made other minor changes in the existing regulations. Clauses were also inserted making the minimum period of training four months in the case of a woman producing a certificate of three years' training as a nurse in a general hospital of not less than 100 beds, or in a Poor-Law institution recognised as a training school for nurses by the Local Government Board, or a certificate of enrolment as a Queen's Nurse. These new regulations were approved by the Privy

Council, and were circulated amongst the institutions recognised as training schools by the Board in December 1915. The regulations are to come into force on July 1, 1916. We are not now concerned with the desirability or otherwise of the changes thus introduced. But we wish to protest strongly against changes of this character being introduced without the heads of institutions engaged in the training of midwifery pupils being afforded an opportunity to express their opinions upon them. The extension of the period of training will involve considerable additional expense to women who desire to become midwives, and this may lead, at any rate for a time, to a serious diminution in the number of candidates. This is specially undesirable at the present time, when the increased demands for women workers in various fields of occupation directly limits the supply. The demands of the Army, for instance, lead many nurses who would otherwise qualify as midwives to enter the Army Nursing Service directly they have obtained their certificates of general training. In our opinion, the time has come when the constitution of the Board should be revised so as to make it more directly representative of those engaged in the training of midwife pupils and in practice as midwives. In any case, the power of the Board to make fundamental alterations in its regulations without any consultation of the recognised midwifery training schools should be withdrawn.

### AN INDICTMENT OF THE BRADFORD CLERGY.

A conspicuous exception to the general revival of Hospital Sunday is to be found at Bradford, where some striking instances of apparent apathy on the part of the clergy and their congregations have been brought forward at the annual meeting of the Bradford Hospital and Convalescent Fund. While the collections at the workshops show an increase of over £300, the sum subscribed by the places of worship again show no increase, and have now fallen to £635. The Lord Mayor confessed himself unable to explain the reason of this. Another speaker remarked that Halifax, a town much smaller than Bradford, had contributed almost double, and the Rev. E. A. Miller remarked that he felt "exceedingly ashamed of the miserable response" from places of worship. With regard, he added, to the number of replies to circulars sent out, he thought they were doing exceedingly well if they got 163 replies to 300 circulars sent to parsons. He had a good deal of correspondence with his brother clergy, but he hardly ever reached so high a percentage as that. He suggested that information relating to the work done by the Fund should be circulated a week before Hospital Sunday. Bradford is growing rich in war-time by leaps and bounds. Are riches destroying all sense of duty? There seems any way no escape from the conclusion that the Bradford clergy and their congregations are gravely neglecting their duty in respect of the claims of Hospital Sunday; they are indicted by one of themselves. How long will they consent to stand in the pillory as unenviable exceptions to the enthusiasm which has made the revival of Hospital Sunday one of the most striking instances of charitable effort in war-time?

### THE FINANCE OF THE INSURANCE ACT.

A very large committee has been appointed by the Treasury to investigate the finances of the National Insurance Act. From such light as is cast by reports of Insurance Committees, approved societies, and similar agencies in the administration of the Act, National Insurance is financially in a bad way. Some of those who have studied the working of it do not hesitate now to speak of it as bankrupt; and, though the war has no doubt accentuated the difficulties of the approved societies, it was quite clearly indicated in these columns two years ago and more that the finance of the Act was thoroughly and radically unsound. The findings and recommendations of the Treasury Committee will be of great interest. The approved societies are very well represented upon it; and the only doubt as to the value of the Committee's labours is that suggested by the large number of those who are to take part in its deliberations. Large committees are rarely productive of good work, and this must be quite one of the largest departmental committees that have been constituted for some time. It will have at least the benefit of an experienced chairman, Sir Gerald H. Ryan, general manager of the Phœnix Assurance Company.

### AN INSTITUTIONAL WILL.

Some very noteworthy bequests to charities and institutions are contained in the will of Mr. Cornelius Surgey, of Hounslow, who died on December 8 last. The following will receive £5,000 each: the Metropolitan Convalescent Institution, Sackville Street; the Home for the Dying, Clapham Common; St. Mary's Hospital, Paddington; the Friedenheim Home for the Dying, Swiss Cottage; the Hospital for Sick Children, Great Ormond Street; Dr. Barnardo's Homes; the Church Army; and the Salvation Army. Various charities receive £1,000 each, among which may be mentioned the Trained Nurses' Annuity Fund, the Harlington Cottage Hospital, and the Hounslow Hospital. Altogether nearly £50,000 out of an estate of £94,313 accrues to various charities.

### MEDICAL MEN AND THE COMPULSION BILL.

During the Committee stage of the Compulsion Bill in the House of Commons, as stated in our Parliamentary news column last week, an amendment was moved by Mr. King to exempt from its provisions all fully qualified medical practitioners, registered dentists, and medical students. Mr. Walter Long refused to accept the amendment, which was negatived without a division. At the same time Mr. Long admitted that medical men ought not to be enlisted as combatant soldiers unless they can really be spared from the Royal Army Medical Corps, which is evidently not the case. With that expression of official opinion few can quarrel; and for our own part we can see neither justice nor reason in the argument that unmarried medical men of under forty should be exempted from the provisions of this Bill when the rest of the male population (of similar state) is included in it. So many doctors of forty and fifty, and even more, are serving their country abroad that the bachelors of military age should be roped in without any delay. We trust there may not be many of them, for the honour of the medical profession; but to our knowledge there are some, for whom compulsion is the only measure. Probably Mr. King's amendment was not due to any special tenderness for the medical profession, but merely part of the wrecking tactics of the irreconcilable faction.

### THE RIGHT TO POSTPONE THE REALISATION OF SECURITIES.

We have already drawn attention to the application of the late Mr. Murray's executor for an order to postpone the sale and realisation of certain securities which formed part of the testator's residuary estate. The case known as Falkiner *v.* Steevens' Hospital, Dublin, and the Attorney-General was again taken before the Court, the Master of the Rolls presiding. The matter concerned bequests amounting to some £22,000 in favour of ten hospitals in Dublin. The plaintiff stated that of the ten hospitals benefiting by the will the only objectors were the governors of Steevens' Hospital. The executor proceeded to administer the estate, but he soon discovered that owing to the war some of the securities were greatly depreciated or unsaleable. In the case of these the executor's view was that it would be bad policy to force a sale. A list of the securities which it was inadvisable to place on the market was submitted, and the Master of the Rolls made an order for the sale of all the securities except the Midland Great Western Railway Preference Stock, which was to be divided in equal shares among the charities.

### DEATH OF TWO MEDICAL KNIGHTS.

On the same day occurred the deaths of two medical knights, Sir Francis Lovell and Sir George Pilkington. The former, who was in his seventy-second year, is best known for his services to tropical medicine and for his tenure during the last thirteen years of the post of Dean of the London School of Tropical Medicine. His previous career had lain almost wholly in the Tropics: he was colonial surgeon in Sierra Leone from 1873 to 1878, Chief Medical Officer of Mauritius from then to 1893, and Surgeon-General of Trinidad and Tobago from 1893 to 1901. So far from being exhausted by this long period of tropical residence, Sir Francis was exceedingly energetic on behalf of the London School, and visited in its cause many of the West India Islands in 1909. He was a Fellow of the Royal College of Surgeons, a C.M.G. (1893), and a Knight Bachelor (1900). Sir George Pilkington was a Guy's man and practised at Southport for many years, afterwards twice representing that constituency in Parliament. He was also twice Mayor of Southport and High Sheriff of Lancashire, and was a director of the Lancashire and Yorkshire Railway Company and of the Manchester and County Bank. His name was originally Coombe; but on his marriage with a Miss Pilkington he assumed her name.

### MORE FRIGHTFULNESS?

According to the *Journal de Genève*, the Germans have now prohibited the exportation of all publications which refer to or describe improvements in medical or surgical treatment originating from the experiences or researches of doctors in the Fatherland. The reason given for this prohibition is that the Allies should not be able to profit in the smallest measure from any progress that is made in German surgery. This proceeding will occasion neither surprise nor dismay among our Allies and ourselves; it is but a minor addition to a long catalogue. But it will be remarkable if this childish incident receives the approval of the medical profession as a whole in Germany. It is said that even books written by Swiss doctors, but published for them in Germany, are also included in this ban.

### MEDICAL LITERATURE AND THE WAR.

A correspondent draws our attention to the effect of the war on medical periodical literature, especially in Germany and France. From the former country, we learn, 206 such papers have failed to be delivered in America: this may be due to their discontinuance, or perhaps partly also to the recent official embargo on their export. Several well-known French medical papers were suspended on the outbreak of war, owing to the mobilisation of their staffs: notably *La Semaine Médicale*, *Le Progrès Médical*, *La Revue de Médecine*, and *La Revue de Chirurgie*. Others continued their existence in a modified form. The *Paris Médical* appears fortnightly instead of weekly; the *Archives Provinciales de Chirurgie* ceased temporarily, but has now recommenced; the *Archives des Maladies du Cœur et du Sang* appears monthly instead of weekly. Several less prominent journals have re-started after long suspension. A leading Italian paper appeared for the last time in December 1915, after an existence of thirty-seven years—namely, the *Giornale Internazionale delle Scienze Mediche*, of Naples. Here in Britain the *Ophthalmic Review* is appearing at irregular intervals instead of monthly; the *British Journal of Dermatology* is to come out quarterly instead of monthly, and the *Medical Magazine*, started in 1892, has now been suspended.

### THE ROYAL FREE HOSPITAL UNIT IN SERBIA.

A member of this unit has contributed to the *Lancet* a most interesting account of the work and adventures of the party organised and led by Mr. and Mrs. James Berry, both of whom are now in the hands of the Austro-German forces. Apparently Mr. Berry found more scope as a sanitary reformer than as a surgeon, and threw himself into this rôle with his accustomed energy and thoroughness. Much of his pioneer work did not command the approval of the Serbian local authorities, who were inclined to be obstructive; apparently the labour of Austrian prisoners was of great value in introducing the elements of decent sanitation in some places. The whole account gives an amusing but distinctly touching account of Mr. Berry's devotion to duty: an anecdote is told of his reply to the offer of an honorary colonelcy in the Serbian Army, "I would rather be appointed official sanitary officer to Vrn Jatchka Banja." When the invasion came, all men of military age who were not doctors were advised to withdraw: Mr. Berry and his wife and Dr. J. B. Christopherson remained and calmly awaited the enemy.

### A DISPENSARY SECRETARY'S LONG RECORD.

It is still a curious fact that the dispensaries survive and prosper, or, in other words, are as much needed now as they were before the unpopular Insurance Act was ever heard of. Thus we find the following clause in the report issued by the committee of the East Grinstead General Dispensary: "As the institution was almost exclusively used by women and children who did not come under the operation of the National Health Insurance Act, it was really needed; and the number of cases attended proved how much it was needed and appreciated." Little further proof of the inadequacy of the Insurance Act is needed. It is interesting to add, as showing the vitality of the dispensary, that Mr. H. A. Perkins, the secretary, has now completed thirty years' work in this office, a record of which he and the committee and subscribers may well be proud. Long service is not the least of the advantages which the voluntary system brings along with it, and it is always pleasant to note examples of such work in our institutional life.

### THE LONDON GENERAL HOSPITALS: A REDUCTION OF "A LA SUITE" STAFFS.

The D.D.M.S. of the London district has initiated a reform which has been long overdue by a drastic reduction of the *personnel* of the *à la suite* staffs. The work will be done in future by the resident staffs under the superintendence of selected members of the visiting staffs (three surgeons and two physicians, except at times of unusual strain). Seniority is to govern the selection of those who will remain on the "active list" at each hospital. There is no doubt in the minds of those who know that the general hospitals of London have been overstaffed as far as the *à la suite* members are concerned; and this measure will both prove economical financially to the Government and will set free a certain number of medical men for civilian and other work. Possibly a similar change may commend itself to those at the head of the medical arrangements for other districts.

### COTTON WASTE REPLACED BY RAGS.

We are in full sympathy with the Sanitary Committee of the Chelmsford Council and the Medical Officer of Health, who have discovered that unclean rags are being issued in place of cotton waste for wiping men's hands in certain factories, and strongly object to it. It appears that cotton waste is now difficult to obtain, so at a "controlled" factory the workers have been supplied with rags instead. No cleansing process was applied to the rags, nor was any inquiry made as to the source from which they were obtained. It is obvious that this penny-wise-pound-foolish policy must be put a stop to somehow; for one can scarcely imagine anything more likely to result in epidemics of disease. It is apparently uncertain exactly what powers the Council has in such a matter, so the Chelmsford authorities, who are most commendably alert in the matter, have written to the Local Government Board asking for advice. Meanwhile they have called the attention of the firms concerned to the dangers to their workmen of the substitution, and have asked them in their own interests to discontinue the use of rags that have not been cleaned and sterilised.

### MENTAL HOSPITAL ATTENDANTS AND WAR SERVICE.

From time to time the columns of The Hospital have borne testimony to the share which the employees of the London County Mental Hospitals Committee have borne in the war, and further striking evidence of this is contained in a report just submitted to the County Council. At the end of 1915 there were on the staff of the Mental Hospitals Committee 2,149 men, of whom 708 were on war service, this number including those who have died on service or are missing. In addition, 113 are on Government or munition work. Under the Derby Group system 34 single men and 340 married men were attested, and 38 single men and 137 married men were rejected. The number of men of military age who have not offered their services under the Derby system stands at the extraordinarily small figure of six single men and ten married men.

### "KITCHENER'S NAVY."

This picturesque title is given by the soldiers to the hospital barges which are used in France to convey the wounded from clearing hospitals to the base, according to an entertaining letter from a sergeant which has been published in the *Middlesex Chronicle*. The writer gives an interesting description of his experience after being wounded, which show the elaborate organisation of the R.A.M.C. "When I was wounded," he writes, "I was taken to the first-aid post in the trenches, from there to the dressing station, also in the trenches, where I saw a doctor who sent me to a field hospital, which was in a church. From the dressing station to the church I was under shrapnel all the way, and was lucky not to get a second dose. Then I was removed in a motor-ambulance to a clearing hospital about five miles away, where I stayed three days. There I was inoculated with anti-tetanus serum as a preventive against lockjaw, which is extremely likely with shell wounds. From here I was transferred to a hospital barge, and had a three days' journey down the canal to Calais, where I

again entered a hospital. There are a large number of these barges in use, and soldiers refer to them as 'Kitchener's Navy.' There is a doctor and two nurses on board each boat, together with a staff of R.A.M.C. orderlies. Everything is splendidly appointed—in fact, quite a floating hospital." The barges are evidently appreciated, and the smooth travelling must be a blessing to many a man to whom even a journey in a motor ambulance is a trying experience.

### EMPIRE HOSPITAL COUNTRY HOME.

Princess Christian has interested herself in providing a country home for patients of the Empire Hospital requiring prolonged treatment or cure, and as a result of Her Royal Highness's sympathy in the matter Cornelia, Lady Wimborne, has generously fitted up her residence, Templeton House, Roehampton, for the purpose. The sum of £3,000 has been subscribed towards the maintenance of the Home by the initiators and supporters of the scheme, and the usual maintenance grant of 3s. per day per head will be allowed by the War Office. The nursing of the Home will be carried out under the superintendence of Miss Mackintosh, the matron of the Empire Hospital. So far, thirteen beds have been arranged for. The control of the medical staff and the allocation of patients will be in the hands of Lord Knutsford's committee, whose group of hospitals, it will be remembered, the Empire Hospital is affiliated with. The Home will be ready for occupation early in February.

### ST. KATHARINE'S AND THE EAST END.

The Poplar Borough Council has lately expressed its gratitude for the work performed by the Royal College of St. Katharine, of which Queen Alexandra is patron, since its establishment in the borough. It is now a year since the College began its work, on the new maternity scheme, in the district. Here three maternity centres have been established, where advice is given by a medical man to expectant and nursing mothers, and to children up to school age, while a dentist also attends weekly. The staff of the College now includes six health visitors, and three more are to be added. The present accommodation of the College at Bromley Hall is to be enlarged by two additional houses, which are to be incorporated in the present institution. The district served by the maternity centres is, of course, so populous that there can hardly be any doubt that the work will continue to grow; and at a time like the present, when the tendency is for local authorities to retrench on socially useful expenditure of this character, it is encouraging to see a charity at work which should be independent of any mistaken calls for economy.

### THE EMPLOYMENT OF CONSUMPTIVES.

An association has been formed at Huddersfield "to secure suitable work for convalescent tuberculous patients, and in general to work in connection with the Public Health Department" in establishing all preventive measures "and to make use of the various organisations represented for the restoration" of individual patients to health and independence. The new society, which is composed of various organisations, is known as the Huddersfield Association for the Assistance of Tuberculosis Patients, and one of their first acts has been to invite the organising secretary of the National Association for the Prevention of Consumption to give an address on the subject of after-care. The immediate problem, which largely resolves itself into inducing employers to take convalescent consumptives into such employment as, with care, may not prove unsuitable for them, is urgent and difficult; for to find a suitable employer is often as hard as to find a suitable occupation. There seems no way of doing this on an adequate scale unless and until an organised public opinion is created to overcome the exaggerated fear of the convalescent consumptive, which has been one of the inevitable results of the anti-phthisis crusade. If the new Huddersfield association can achieve this, and at the same time aid the convalescent to live such an out-door life as may be possible in his own home, before the habits acquired in the sanatorium be forgotten, it should be a very useful body. Advice on home-made shelters and such homely, practical points on the one hand, and the getting of local employers generally to feel that they have a common responsibility towards the convalescent consumptive on the other: these are the two practical lines on which the new society should work.

### THIS WEEK'S DRUG MARKET.

A revival in quinine and a further considerable advance in the price of cocaine are the main events that have happened in the drug market since the last report was written. The rise in the value of quinine is due this time to a substantial increase in the price of the raw material—cinchona bark—and some large buying of the usual Continental brands of quinine have been made at advancing rates. Those whose stocks of quinine have run low should, however, make careful inquiries before purchasing in any considerable quantity. The further advance in the price of cocaine is difficult to account for, and the opinion is held in some quarters that the present value is inflated; caution should be exercised in making purchases of this drug as it is quite probable that the high value will not be maintained. Potassium bromide shows no sign of any impending decline in value; indeed, all indications point to the probability of a further advance. It is interesting, if somewhat disconcerting, to note that in Germany, where supplies of bromide and potash are abundant, potassium bromide can be bought at less than one-thirtieth the price that has to be paid both here and in America. Salol has become much scarcer, and the price has advanced. Pilocarpine hydrochloride is not readily obtainable, although the nitrate appears to be in fair supply. Potassium permanganate is again dearer. Citric acid has an advancing tendency in value, mainly as a result of large orders from Russia; tartaric acid is also inclining upwards in price. Hexamine is one of the few drugs that are cheaper; this is due to the arrival of supplies from the United States.

